# Healthcare professionals’ knowledge, attitudes and practices in thromboelastography application

**DOI:** 10.3389/fmed.2025.1645570

**Published:** 2025-10-07

**Authors:** Yifei Jia, Xiangyu Zhu, Xiaoling Yu, Weihong Xu, Weidong Wu, Jianfeng Ye, Liping Wang

**Affiliations:** The Clinical Laboratory of Shanghai Tongren Hospital, Shanghai, China

**Keywords:** thromboelastography, healthcare professionals, knowledge, attitudes, practices, cross-sectional study

## Abstract

**Objective:**

This study aims to investigate the knowledge, attitudes, and practices (KAP) of healthcare professionals regarding thromboelastography (TEG).

**Methods:**

A cross-sectional study was conducted at Tongren Hospital in Shanghai from January to February 2025. Demographic data, knowledge, attitude, and practices scores, were collected and evaluated via a self-developed questionnaire.

**Results:**

A total of 218 valid responses were included in the analysis. Of the participants, 130 (59.63%) were physicians, and 88 (40.37%) were nurses. TEG-related training had been received by 149 (68.35%) of the participants. The mean proficiency score for TEG use was 5.83 ± 2.90 (range: 0–10). The mean scores for knowledge, attitude, and practice were 8.10 ± 2.44 (range: 0–12), 30.81 ± 7.11 (range: 8–40), and 28.92 ± 8.87 (range: 8–40), respectively. Significant positive correlations were observed between knowledge and attitude (*r* = 0.1722, *p* = 0.0109) and between attitude and practice (*r* = 0.6945, *p* < 0.001). The structural equation model revealed that knowledge (*β* = 0.76, *p* < 0.001) directly influenced attitude, and attitude (*β* = 1.10, *p* < 0.001) directly influenced practice. Additionally, years of practice (*β* = 0.84, *p* = 0.003) and frequent use of TEG (*β* = −0.79, *p* = 0.024) were found to impact knowledge, which in turn affected attitude (*β* = 0.76, *p* < 0.001).

**Conclusion:**

The majority of healthcare professionals demonstrated inadequate knowledge, positive attitudes, and optimal practices regarding TEG. Enhanced training programs focused on TEG could improve proficiency and optimize its clinical application, especially for professionals with fewer years of experience.

## Introduction

Thromboelastography (TEG), developed in 1948 by Dr. Hellmut Hartert at Heidelberg University, provides a dynamic and comprehensive assessment of the coagulation process (1). TEG provides a physiologically accurate assessment of the coagulation system and has been effectively utilized as a rapid point-of-care test to assess hypercoagulable, hypocoagulable, and rebalanced coagulation states. This enables clinicians to evaluate blood transfusion requirements, determine the need for anticoagulation, and select the appropriate anticoagulant therapy (2).

TEG has demonstrated various applications across multiple clinical specialties, including trauma care, obstetrics, orthopedics, surgical ICU, cardiovascular surgery, and transplantation. For example, in trauma and cardiac surgery, TEG helps predict bleeding risks and transfusion needs. It also serves as a valuable tool for monitoring anticoagulation therapy during orthopedic procedures, with studies showing TEG parameters can effectively guide anticoagulation management and reduce bleeding complications by 15%–20% (3). TEG has demonstrated significant utility in predicting hypercoagulability, with research reporting elevated maximum amplitude (MA) values (65.2 ± 7.3 mm vs. 58.7 ± 5.4 mm in controls, *p* < 0.001) in acute kidney injury patients (4). In transplant patients, abnormal TEG parameters (specifically R-time <5 min and MA > 70 mm) have been associated with a 2.3-fold increased risk of coronary events (5). Furthermore, studies have demonstrated TEG’s effectiveness in neurosurgery and abdominal surgeries by identifying hypercoagulable states in pregnancy complications with significantly decreased reaction time (R-time) values (5.2 ± 1.1 min vs. 6.8 ± 1.4 min in normal pregnancies, *p* < 0.05) and increased maximum amplitude (MA) values (68.7 ± 4.2 mm vs. 62.3 ± 3.9 mm, *p* < 0.05) (6). Despite its advantages, inadequate knowledge and improper use of TEG can contribute to poor clinical outcomes. For instance, inadequate assessment of the hematologic system or failure to recognize risk factors for bleeding can result in uncontrolled bleeding, empiric administration of blood products, and increased exposure to bloodborne pathogens (7).

According to the Knowledge, Attitude, and Practice (KAP) theory, knowledge forms the foundation of behavior change, while attitudes and beliefs act as the driving force for these changes (8). The KAP model divides behavior change into three distinct stages: acquiring knowledge, forming attitudes and beliefs, and developing practices or behaviors (9). Importantly, cognitive changes induced by knowledge acquisition do not directly translate into behavioral change; instead, a shift in perception must occur first, leading to subsequent behavior modification (10). This sequence in the KAP model is essential for modifying physicians’ clinical practice patterns (11). Despite TEG’s significant clinical value, challenges remain regarding healthcare professionals’ knowledge, attitudes, and practices in its use. Substantial differences in the understanding and application of TEG among healthcare professionals can lead to inconsistencies in anticoagulant and antithrombotic treatments, potentially impacting patient outcomes (12).

At present, most research on TEG has focused on its clinical applications, with limited studies investigating healthcare professionals’ KAP regarding TEG (13, 14). Therefore, this study aims to assess the knowledge, attitudes, and practices related to TEG among healthcare professionals at a tertiary hospital in China.

## Materials and methods

### Study design and participants

This cross-sectional study was conducted at Tongren Hospital in Shanghai from January to February 2025, targeting healthcare professionals. This study was approved by the Medical Ethics Committee of Shanghai Tong Ren Hospital (Approval No.: K2024-097-02). Written informed consent was obtained from all participants. Inclusion criteria were healthcare professionals, specifically physicians with valid medical licenses and registered nurses with legal certification to practice. Exclusion criteria were: (1) physicians or nurses not actively engaged in clinical practice; and (2) individuals in training positions, such as interns, rotating doctors, or advanced trainees.

### Questionnaire

The questionnaire was designed based on established guideline (15) and relevant literature (16, 17). The questionnaire has not been previously published nor officially recommended by a scientific society. It was not adapted or translated from international sources; therefore, no forward–backward translation procedure was required. However, its development followed expert review and a pilot test to ensure content validity and internal consistency. After the initial draft, the questionnaire was reviewed by a senior expert with 40 years of experience in the field of the endocrinology department. Revisions were made based on the expert’s feedback, including adjustments and the removal of ambiguous or redundant items. A pilot test, conducted with 30 participants, yielded a Cronbach’s *α* of 0.9515 overall, with 0.8963 for the knowledge section, 0.9218 for the attitude section, and 0.9810 for the practice section, indicating strong internal consistency. A pilot test, conducted with 30 participants, yielded a Cronbach’s *α* of 0.9515 overall, with 0.8963 for the knowledge section, 0.9218 for the attitude section, and 0.9810 for the practice section, indicating strong internal consistency. Confirmatory factor analysis (CFA) indicated good model fit. The Kaiser-Meyer-Olkin (KMO) value was 0.945 (*p* < 0.001), supporting sampling adequacy.

The final questionnaire, written in Chinese, included four sections: demographic data (including gender, age, education level, occupation, title, years of work, hospital level, which refers to the grading of the hospital in which the participant was currently working, classified as tertiary, secondary, primary, or private according to the official hospital classification system in China, TEG training experience, and self-rated proficiency in TEG usage), knowledge section, attitude section, and practice section. Proficiency in TEG use was assessed based on participants’ self-reported scores. Each participant rated their familiarity with TEG on a scale from 0 (“completely unfamiliar”) to 10 (“very familiar and proficient”), with responses restricted to whole integers. The knowledge dimension included 12 questions, with 1 point for correct answers and 0 points for unclear or incorrect answers, resulting in a score range of 0–12. The attitude dimension consisted of 8 questions using a 5-point Likert scale, with scores ranging from 5 (strongly agree) to 1 (strongly disagree), resulting in a score range of 8–40. The practice dimension included 8 questions also using a 5-point Likert scale, with scores ranging from 5 (always) to 1 (never), resulting in a score range of 8–40. A threshold of ≥70.0% was set to define adequate knowledge, positive attitudes, and optimal behaviors (18, 19). This threshold is consistent with recent KAP studies that define adequacy/favorability/optimality at ≥70% of the maximum score for each domain, providing a pragmatic and interpretable standard across settings. Representative applications of the 70% cutoff have been reported in pediatric healthcare KAP (20), oral-health KAP (21).

### Questionnaire distribution and quality control

Data collection was conducted online using an electronic questionnaire. The questionnaire was distributed via Wenjuanxing,^1^ and participants were recruited through phone calls, WeChat messages, WeChat Moments, and in-person communication. The questionnaire was distributed via Wenjuanxing, with links provided to participants through WeChat and in person at hospitals that conduct thromboelastography testing. There were no restrictions on the departments involved. Participants were contacted via phone, WeChat, or in person to obtain their consent. If participants had any difficulty understanding the questionnaire, a dedicated researcher was available to explain the content of the survey. Exclusion Criteria: (1) Questionnaire completion time <90 s; (2) Significant logical inconsistencies between answers to different questions, such as inconsistencies or contradictions; this criterion was intended to identify responses with low reliability or inattentive answering behavior; (3) Incomplete responses that prevented comprehensive information collection or effective analysis.

### Sample size

The minimum sample size was estimated based on the method of calculating five times the total number of demographic and KAP items in the questionnaire, as recommended for survey studies (22). Since the questionnaire consisted of 38 items, the estimated minimum sample size was 190. To account for a potential 10% rate of invalid responses, the adjusted minimum sample size was set at 209.

### Statistical methods

Data were analyzed using STATA 17.0 (Stata Corporation, College Station, TX, USA). The normality of continuous variables was tested using the Kolmogorov–Smirnov test. Continuous variables with normal distribution were expressed as mean ± standard deviation (SD) and analyzed using t-tests or analysis of variance (ANOVA). Skewed data were expressed as median (range) and analyzed using the Wilcoxon-Mann–Whitney U test or Kruskal-Wallis ANOVA. Categorical variables were expressed as n (%). Spearman correlation analysis was used to assess the correlations between KAP scores. The univariate and multivariate logistic regression analyses to examine the independent influence factors of knowledge scores. Structural Equation Modeling (SEM) was used to test the hypotheses that knowledge (H1) directly affects attitudes, knowledge (H2) directly affects behavior, and knowledge (H3) indirectly affects behavior through attitudes. In addition, separate subgroup analyses were conducted for physicians and nurses. Model fit was assessed using multiple indices: Root Mean Square Error of approximation (RMSEA), standardized root mean square residual (SRMR), incremental fit index (IFI), Tucker-Lewis index (TLI), and comparative fit index (CFI). A two-sided *p*-value <0.05 was considered statistically significant.

## Results

### Demographic information of the participants

Out of 228 submitted questionnaires, 10 were excluded due to completion times under 90 s, resulting in 218 valid responses and an effective response rate of 95.61%. The mean age of participants was 36.17 ± 6.11 years. Of the participants, 136 (62.39%) were female, 112 (51.38%) held a master’s degree or higher, 130 (59.63%) were physicians, 105 (48.17%) had a junior job rank, 149 (68.35%) had received TEG-related training in their hospitals, and 133 (61.01%) frequently used TEG in clinical practice.

The average proficiency score in TEG use was 5.83 ± 2.90 (range: 1–10). The mean scores for knowledge, attitude, and practice were 8.10 ± 2.44 (range: 0–12), 30.81 ± 7.11 (range: 8–40), and 28.92 ± 8.87 (range: 8–40), respectively. Knowledge scores significantly differed by age (*p* < 0.001), job rank (*p* = 0.039), years of medical practice (*p* < 0.001), and frequent TEG use in clinical practice (*p* = 0.038). Attitude scores were significantly associated with TEG-related training (*p* = 0.046). Practice scores showed significant differences based on position (*p* = 0.031), job rank (*p* = 0.003), and years of medical practice (*p* = 0.035) (Table 1).

**Table 1:** Demographic information and KAP scores of the participants.

*N* = 218	*N* (%)	Knowledge score		Attitude score		Practice score	
		Mean ± SD	*P*	Mean ± SD	*P*	Mean ± SD	*P*
Total score		8.10 ± 2.44		30.81 ± 7.11		28.92 ± 8.87	
Age (years old)	36.17 ± 6.11		**<0.001**		0.648		0.120
≤30	34(15.6)	8.20 ± 2.02		30.14 ± 7.65		29.76 ± 9.13	
31–40	156(71.56)	7.82 ± 2.44		30.87 ± 7.16		29.07 ± 8.88	
>40	28(12.84)	9.53 ± 2.51		31.25 ± 6.27		27.03 ± 8.51	
Gender			0.923		0.104		0.956
Male	82(37.61)	8.28 ± 2.19		30.28 ± 6.98		29.09 ± 8.66	
Female	136(62.39)	7.99 ± 2.58		31.13 ± 7.19		28.81 ± 9.01	
Education level			0.397		0.650		0.943
Bachelor’s degree or below	106(48.62)	8.16 ± 2.71		30.90 ± 7.29		28.80 ± 9.05	
Master’s degree or above	112(51.38)	8.03 ± 2.17		30.72 ± 6.96		29.03 ± 8.72	
Occupation			0.316		0.052		**0.031**
Doctor	130(59.63)	8.04 ± 2.33		30.48 ± 6.79		28.42 ± 8.40	
Nurse	88(40.37)	8.18 ± 2.61		31.29 ± 7.56		29.65 ± 9.50	
Professional rank			**0.039**		0.100		**0.003**
No rank	48(22.02)	7.85 ± 1.82		31.06 ± 7.40		30.12 ± 8.50	
Junior	105(48.17)	7.85 ± 2.45		30.65 ± 7.47		29.66 ± 9.25	
Intermediate	54(24.77)	8.66 ± 2.71		31.27 ± 6.63		27.61 ± 8.20	
Senior (including associate senior)	11(5.05)	8.72 ± 3.03		28.90 ± 4.43		23 ± 7.61	
Years of medical practice	9.08 ± 7.27		**<0.001**		0.904		**0.035**
≤3 years	30(15.63)	8.03 ± 1.90		31.63 ± 5.74		31.53 ± 7.10	
4–10 years	108(56.25)	7.53 ± 2.22		30.16 ± 7.77		28.97 ± 9.45	
11–20 years	37(19.27)	9.05 ± 3.24		31.27 ± 5.88		25.97 ± 9.08	
More than 20 years	17(8.85)	10.1 ± 1.62		32.11 ± 5.98		28.29 ± 7.23	
Hospital level			0.073		0.971		0.362
Tertiary hospital	110(50.46)	8.34 ± 2.81		31.10 ± 6.98		28.36 ± 9.22	
Secondary hospital	58(26.61)	8.03 ± 2.11		30.63 ± 7.43		29.86 ± 8.75	
Primary hospital	29(13.3)	7.58 ± 1.82		30.20 ± 7.45		28.79 ± 8.37	
Private hospital	21(9.63)	7.71 ± 1.87		30.57 ± 6.83		29.42 ± 8.27	
Has your hospital provided training related to thromboelastography			0.143		**0.046**		0.529
Yes	149(68.35)	8.36 ± 2.16		31.26 ± 7.08		29.26 ± 8.64	
No	69(31.65)	7.53 ± 2.90		29.82 ± 7.12		28.17 ± 9.35	
Frequently use thromboelastography in clinical practice			**0.038**		0.226		0.182
Yes	133(61.01)	8.42 ± 2.26		31.19 ± 7.14		29.61 ± 8.62	
No	85(38.99)	7.58 ± 2.63		30.21 ± 7.06		27.83 ± 9.17	
Proficiency in using thromboelastography	5.83 ± 2.90						

Hospital level classification follows the official grading system in China: tertiary (highest level), secondary, primary, and private hospitals. Classification reflects the participant’s current primary workplace. The result was statistically significant (*P* < 0.05).

### Knowledge, attitude, and practice

In the knowledge section, the three most commonly selected “unsure” responses were: “Some low-temperature blood specimens analyzed by thromboelastography can reflect the true coagulation function status” (K14) at 5.96%, “Thromboelastography can assess endothelial function” (K13) at 7.8%, and “An increased R-value indicates higher coagulation factor activity and a hypercoagulable state” (K4) at 27.98% (Table 2). In the attitude dimension, 8.72% strongly disagreed that TEG is a valuable tool for diagnosing and treating thrombotic diseases (A2), 8.26% strongly disagreed that healthcare professionals recognize the potential advantages of TEG (A6), and 8.26% strongly disagreed that more training is needed to better utilize TEG (A8) (Table 3). In the practice dimension, 12.39% reported being completely unfamiliar with TEG application across patient populations (P6), 11.01% never discussed TEG with colleagues (P8), and 10.09% never used TEG in daily clinical practice to assess disease progression (P1) or to guide treatment plans (P2) (Table 4).

**Table 2:** Knowledge dimension distribution.

Items	Accuracy rate, *N* (%)
1. Thromboelastography reflects the dynamic changes in blood coagulation.	153 (70.18)
2. Thromboelastography provides complete information from coagulation initiation to platelet aggregation, fibrin strand formation, clot growth, maximum clot formation, clot degradation, and dissolution.	157 (72.02)
3. Parameters of thromboelastography include Coagulation Reaction Time (R), Clot Formation Time (K), Clot Formation Rate (Angle), Maximum Amplitude (MA), Comprehensive Coagulation Index (CI), LY30, and EPL.	146 (66.97)
4. An increased R value indicates higher coagulation factor activity and a hypercoagulable state.	61 (27.98)
5. An increased K value indicates reduced fibrinogen function and a hypocoagulable state.	150 (68.81)
6. An increased Angle indicates strong fibrinogen function and a hypercoagulable state.	128 (58.72)
7. The MA value primarily reflects platelet function.	142 (65.14)
8. LY30/EPL primarily reflects fibrinogen function.	147 (67.43)
9. Tromboelastography can evaluate the efficacy of aspirin and clopidogrel in patients with coronary artery disease.	155 (71.1)
10. Thromboelastography can monitor the anticoagulant efficacy of heparin and low molecular weight heparin.	142 (65.14)
11. Thromboelastography can perform coagulation monitoring for postpartum hemorrhage and guide component transfusion.	136 (62.39)
12. Thromboelastography can predict the risk of deep vein thrombosis.	139 (63.76)
13. Thromboelastography can assess endothelial function.	17 (7.8)
14. Some low-temperature blood specimens analyzed by thromboelastography can reflect the true coagulation function status.	13 (5.96)

**Table 3:** Attitude dimension distribution.

*N* (%)	Very positive	Positive	Neutral	Negative	Very negative
1. What is your general attitude and that of your colleagues towards the clinical application of thromboelastography?	111 (50.92)	78 (35.78)	27 (12.39)	2 (0.92)	/
2. Do you believe that thromboelastography is a valuable tool that can assist in the diagnosis and treatment of thrombotic diseases?	71 (32.57)	85 (38.99)	31 (14.22)	12 (5.5)	19 (8.72)
3. Do you feel confident and assured when interpreting thromboelastography results?	60 (27.52)	84 (38.53)	41 (18.81)	22 (10.09)	11 (5.05)
4. When faced with thromboelastography results, do you tend to include them as one of the factors in clinical decision-making?	62 (28.44)	86 (39.45)	41 (18.81)	18 (8.26)	11 (5.05)
5. Do you believe that thromboelastography is more accurate and reliable for thrombotic risk assessment compared to traditional methods?	68 (31.19)	86 (39.45)	35 (16.06)	14 (6.42)	15 (6.88)
6. Do you think that healthcare professionals generally recognize the potential advantages of thromboelastography in preventing and managing related diseases?	55 (25.23)	93 (42.66)	42 (19.27)	10 (4.59)	18 (8.26)
7. Are you willing to actively promote the application and adoption of thromboelastography in clinical practice?	59 (27.06)	93 (42.66)	37 (16.97)	15 (6.88)	14 (6.42)
8. Do you think healthcare professionals need more training and guidance to better utilize thromboelastography for disease management?	65 (29.82)	92 (42.2)	34 (15.6)	9 (4.13)	18 (8.26)

**Table 4:** Practice dimension distribution.

*N* (%)	Always	Often	Sometimes	Occasionally	Never
1. Do you frequently use thromboelastography in your daily clinical practice to assess disease progression?	59 (27.06)	76 (34.86)	44 (20.18)	17 (7.8)	22 (10.09)
2. Do you follow the results of thromboelastography to develop treatment plans?	64 (29.36)	71 (32.57)	42 (19.27)	19 (8.72)	22 (10.09)
3. Do you monitor and record the effects and changes of thromboelastography during the patient’s treatment process?	59 (27.06)	73 (33.49)	47 (21.56)	23 (10.55)	16 (7.34)
4. Do you combine thromboelastography results with other coagulation tests (e.g., PT, APTT)?	69 (31.65)	68 (31.19)	40 (18.35)	24 (11.01)	17 (7.8)
5. Are you able to accurately interpret and apply the various parameters when evaluating thromboelastography results?	61 (27.98)	76 (34.86)	34 (15.6)	28 (12.84)	19 (8.72)
6. Are you familiar with the application of thromboelastography in different patient populations (e.g., surgical patients, trauma patients)?	60 (27.52)	73 (33.49)	42 (19.27)	16 (7.34)	27 (12.39)
7. Do you regularly update your knowledge about thromboelastography and stay informed about the latest research and guidelines?	64 (29.36)	72 (33.03)	40 (18.35)	27 (12.39)	15 (6.88)
8. Do you frequently discuss and share experiences with other healthcare professionals regarding the use of thromboelastography in diagnosis and treatment?	60 (27.52)	73 (33.49)	38 (17.43)	23 (10.55)	24 (11.01)

Based on the predefined cutoffs (≥70% of the maximum score for each dimension), 64 participants (29.36%) achieved adequate knowledge, while 154 (70.64%) had inadequate knowledge. For attitudes, 169 participants (77.52%) demonstrated positive attitudes, and 49 (22.48%) had less positive attitudes. Regarding practices, 149 participants (68.35%) achieved optimal practices, whereas 69 (31.65%) reported suboptimal practices (Table 5).

**Table 5:** Distribution of participants by knowledge, attitude, and practice (KAP) classification based on predefined cutoffs.

*N* (%)	K	A	P
≥ Cutoff value	64 (29.36%)	169 (77.52%)	149 (68.35%)
< Cutoff value	154 (70.64%)	49 (22.48%)	69 (31.65%)
Total	218 (100%)	218 (100%)	218 (100%)

### Correlations between KAP

Correlation analysis revealed significant positive correlations between knowledge and attitude (*r* = 0.1722, *p* = 0.0109) and between attitude and practice (*r* = 0.6945, *p* < 0.001). However, no significant correlation was observed between knowledge and practice (*r* = 0.1150, *p* = 0.0903), indicating that higher knowledge does not necessarily translate into improved practice behaviors (Table 6).

**Table 6:** Correlation analysis.

Items	Knowledge	Attitude	Practice
Knowledge	1		
Attitude	0.1722 (*P* = 0.0109)	1	
Practice	0.1150 (*P* = 0.0903)	0.6945 (*P* < 0.001)	1

### Multivariate logistic regression analysis for knowledge dimension

Univariate and multivariate logistic regression analyses were performed to identify factors associated with good knowledge scores (≥70% cutoff, ≥9.8 points). In univariate analysis, age ≤30 years (OR: 5.023, 95% CI: 1.677–15.042, *p* = 0.004) and 31–40 years (OR: 4.636, 95% CI: 2.000–10.745, *p* < 0.001) were associated with higher odds of good knowledge compared with >40 years. However, these associations were not significant in the multivariate model. In contrast, years of clinical experience remained significant, with ≤3 years (OR: 18.325, 95% CI: 2.458–136.641, *p* = 0.005) and 4–10 years (OR: 21.263, 95% CI: 3.382–133.696, *p* = 0.001) showing markedly higher odds compared with >20 years. Working in a secondary public hospital was also significantly associated with lower odds of good knowledge (OR: 0.145, 95% CI: 0.024–0.854, *p* = 0.033) compared with private hospitals. Other factors, including gender, education level, position, professional rank, TEG training, frequency of TEG use, and self-rated proficiency, were not significantly associated with knowledge scores in the multivariate model (Table 7).

**Table 7:** Regression analysis for knowledge dimension.

Items	Univariate logistic regression		Multivariate logistic regression	
	OR (95%CI)	*P*	OR (95%CI)	*P*
Age (years old)				
≤30	5.023 (1.677–15.042)	0.004	0.363 (0.066–2.008)	0.245
31–40	4.636 (2.000–10.745)	<0.001	0.560 (0.139–2.256)	0.415
>40	ref		ref	
Gender				
Male	1.007 (0.552–1.838)	0.982		
Female	ref			
Education level				
Bachelor’s degree or below	0.592 (0.329–1.068)	0.081		
Master’s degree or above	ref			
Occupation				
Doctor	1.334 (0.740–2.406)	0.338		
Nurse	ref			
Professional rank				
No rank	2.803 (0.716–10.968)	0.139		
Junior	2.812 (0.789–10.027)	0.111		
Intermediate	1.042 (0.283–3.832)	0.951		
Senior (including Associate Senior)	ref			
Years of medical practice				
≤3 years	9.600 (2.429–37.942)	0.001	18.325(2.458–136.641)	0.005
4–10 years	11.242 (3.542–35.679)	<0.001	21.263(3.382–133.696)	0.001
11–20 years	1.829 (0.535–6.252)	0.336	2.066(0.452–9.438)	0.349
More than 20 years	ref		ref	
Hospital level				
Tertiary hospital	0.177 (0.039–0.800)	0.024	0.229(0.043–1.215)	0.083
Secondary hospital	0.254 (0.053–1.212)	0.086	0.145(0.024–0.854)	0.033
Primary hospital	0.658 (0.109–3.977)	0.648	0.344(0.047–2.544)	0.296
Private hospital	ref		ref	
Has your hospital provided training related to thromboelastography				
Yes	0.878 (0.466–1.655)	0.688		
No	ref			
Frequently use thromboelastography in clinical practice				
Yes	0.687 (0.372–1.267)	0.229		
No	ref			
Proficiency in using thromboelastography	0.927 (0.836–1.028)	0.150		

### Structural equation model

**Figure 1:**
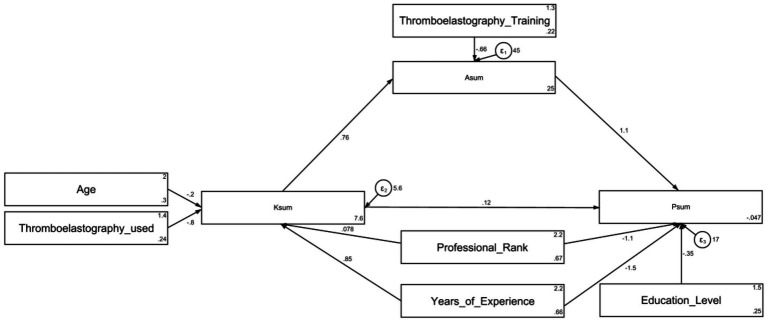
Structural Equation Model (SEM) depicting the associations among demographic variables, and the knowledge (Ksum), attitude (Asum), and practice (Psum) scores related to thromboelastography. Rectangles represent observed variables (e.g., age, training, scores). Circles represent error terms (*ε*₁, ε₂, ε₃). Single-headed arrows represent standardized regression paths; values are standardized coefficients.

The SEM model demonstrated that years of medical practice (*β* = 0.84, *p* = 0.003) and frequent TEG use (*β* = −0.79, *p* = 0.024) directly impacted knowledge. Knowledge (*β* = 0.76, *p* < 0.001) directly affected attitude. Attitude (*β* = 1.10, *p* < 0.001), job rank (*β* = −1.11, *p* = 0.008), and years of practice (*β* = −1.52, *p* = 0.001) directly affected practice. Additionally, years of practice (*β* = 0.64, *p* = 0.018) indirectly impacted attitude, while knowledge (*β* = 0.84, *p* < 0.001), department (*β* = −1.26, *p* < 0.001), and years of practice (*β* = 0.81, *p* = 0.018) indirectly affected practice (Supplementary Table S1 and Figure 1). The SEM demonstrated excellent model fit (RMSEA: 0.003, SRMR: 0.033, TLI: 1.000, CFI: 1.000) (Supplementary Table S2). Multiple group SEM analyses were conducted for physicians and nurses. In physicians, Knowledge significantly predicted Attitude (*β* = 0.690, *p* = 0.007) and Attitude significantly predicted Practice (*β* = 1.064, *p* < 0.001), but Knowledge did not directly predict Practice (*β* = −0.022, *p* = 0.883) (Supplementary Figure S1). In nurses, Knowledge significantly predicted Attitude (*β* = 0.715, *p* = 0.015), Attitude significantly predicted Practice (*β* = 1.096, *p* < 0.001), and the Knowledge → Practice path approached significance (*β* = 0.366, *p* = 0.054) (Supplementary Figure S2).

## Discussion

This study revealed that the majority of healthcare professionals exhibited insufficient knowledge, generally positive attitudes, and optimal practices regarding TEG. Enhancing targeted training programs on TEG, especially for those with fewer years of practice, may improve proficiency and clinical application, ultimately benefiting patient care.

Our findings indicate that while healthcare professionals showed a generally positive attitude toward TEG, their knowledge and practical application remain limited. This aligns with prior studies that have documented gaps in clinicians’ understanding and use of TEG knowledge and utilization among healthcare professionals, which may contribute to suboptimal clinical outcomes (12). These moderate KAP scores suggest that an insufficient understanding of TEG may contribute to the suboptimal management of related conditions, such as thrombotic complications, in certain clinical scenarios. While TEG can help identify hypercoagulable states, it is not the primary tool for long-term thrombosis risk assessment. Clinical decision-making should remain grounded in comprehensive patient evaluation, including history-taking and conventional coagulation tests. Improving healthcare professionals’ knowledge and usage of TEG is crucial for better patient outcomes.

In examining the relationship between KAP, both the correlation analyses and SEM results highlight significant interactions between knowledge, attitudes, and practices, providing key insights into the role of various factors. The positive correlation between knowledge and attitude is consistent with findings in other studies, suggesting that an increase in knowledge can enhance attitudes toward clinical tools (23, 24). Similarly, the correlation between attitude and practice underscores the importance of fostering a positive outlook to promote better clinical implementation. Although the KAP framework traditionally posits that knowledge directly influences both attitudes and practices, our SEM findings indicated that knowledge significantly influenced attitudes (*β* = 0.76, *p* < 0.001) but did not directly predict practice (*r* = 0.1150, *p* = 0.0903). This aligns with evidence from other healthcare domains suggesting that attitudinal change often mediates the translation of knowledge into behavior (11, 24). In the context of TEG, this may reflect the complexity of integrating viscoelastic testing into routine decision-making, which requires not only technical knowledge but also confidence, perceived utility, and supportive institutional protocols. This theoretical nuance suggests that interventions aiming to enhance TEG use should address both knowledge and attitude concurrently, rather than focusing solely on informational training.

The SEM model further enriches this understanding by showing that variables such as years of medical practice and frequent use of TEG in clinical settings play a pivotal role in shaping knowledge. Notably, subgroup SEM analyses indicated that the Knowledge influence Attitude and Attitude influence Practice pathways were consistent across physicians and nurses, but the magnitude of these effects differed. Nurses showed a stronger potential direct link between knowledge and practice, which approached statistical significance. This may reflect differences in scope of practice, autonomy in TEG interpretation, and integration into routine workflows. Professionals with more experience or who regularly use TEG tend to have higher knowledge scores. This is likely because frequent exposure and hands-on use of TEG allow healthcare professionals to refine their understanding and confidence in applying it (22, 25). These factors contribute not only to better knowledge but also shape more favorable attitudes, suggesting that consistent exposure to TEG may gradually build both confidence and clinical integration. The SEM results offered additional insights. Years of practice and frequent clinical use of TEG were positively associated with higher knowledge scores. These findings point to a feedback loop where professionals with higher knowledge tend to develop more favorable attitudes, which in turn leads to better clinical practice.

The differences observed across variables such as job rank and years of practice further emphasize the importance of tailored interventions. Similar findings have been reported in other KAP studies among healthcare professionals. Previous research found that clinicians’ experience levels significantly influenced their knowledge application in evidence-based practice, with more experienced practitioners showing better integration of new technologies (26). Similarly, studies reported that nurses with advanced ranks and longer years of service demonstrated better knowledge and practices in point-of-care testing, suggesting that experience-based training approaches might be more effective than standardized programs (27). In the field of hemostasis management, researchers observed that anesthesiologists with different years of experience showed varying levels of adherence to transfusion protocols, with those in intermediate positions requiring more targeted education (28). Less experienced professionals, or those in junior positions, exhibited lower scores in both attitude and practice. In contrast, junior professionals or those with fewer years of experience may lack both the confidence and authority to apply complex diagnostics such as TEG (29). The SEM supports this by showing that both job rank and years of experience directly affect practice, indicating that professionals in more senior positions or with more years in the field may have greater autonomy and exposure to TEG in practice, thereby reinforcing better clinical behaviors. Additionally, age and TEG-related training were associated with higher scores in knowledge and attitude. These findings align with several studies in medical education literature. Research on point-of-care diagnostics has demonstrated that structured training programs significantly improve healthcare professionals’ knowledge retention and clinical application (28). A study on viscoelastic testing in critical care settings revealed that professionals who received formal training scored 40% higher on knowledge assessments than their untrained counterparts (30). Similarly, the positive association between age and knowledge scores observed in our study reflects the cumulative effect of clinical experience, consistent with findings from studies on other specialized diagnostic tools, where older healthcare providers demonstrated more comprehensive understanding of complex parameters and their clinical implications. However, unlike some studies that found a potential knowledge plateau with very advanced age, our results suggest a continuous improvement trend, possibly due to the relatively recent introduction of TEG technology in many clinical settings. Addressing these demographic disparities through targeted training programs could help improve the application of TEG in clinical settings.

The distribution of knowledge scores reveals that while most participants had a sound understanding of core TEG parameters, significant gaps remain in areas like endothelial function and fibrinogen evaluation. To address these gaps, targeted training programs focusing on these areas are recommended, incorporating module-based learning and practical case simulations across varied patient populations (31, 32). E-learning platforms and periodic assessments should also be implemented to ensure continuous knowledge updates.

Although the majority of participants were confident in TEG’s clinical application, some hesitancy persisted. Institutions could consider forming TEG-focused committees, incorporating regular interdisciplinary discussions, and promoting peer mentoring between experienced and junior professionals would help. Integrating TEG into decision-making protocols and creating a TEG committee to review outcomes and promote best practices could further enhance confidence (33–35).

In the practice dimension, low scores were recorded for the routine use of TEG in clinical decisions and experience-sharing among colleagues. Encouraging regular interdisciplinary case discussions involving TEG may offer a practical way to bridge the gap between knowledge and daily clinical use. These discussions could be scheduled as part of weekly or monthly departmental meetings, where complex cases involving TEG use are reviewed and strategies for improving its clinical application are explored. Furthermore, the creation of a clinical practice guideline specific to TEG use could help ensure consistency in its application (15, 36). This guideline should be disseminated through mandatory training sessions for all relevant clinical staff, with follow-up audits to monitor adherence. For professionals at different job ranks or levels of experience, tailored interventions should be implemented. For instance, junior staff could benefit from hands-on workshops focused on interpreting and applying TEG results, while senior clinicians might engage in advanced courses that integrate TEG results with other diagnostic tools to optimize patient care (36–38).

This study has several limitations. First, the use of self-reported questionnaires may introduce response bias, as participants may overestimate their knowledge or practices. Second, due to the online distribution of the questionnaire via social media, some respondents may have been from outside the study hospital, and their origins could not be verified. This may have introduced selection bias. Third, the cross-sectional design of the study prevents the determination of causality between the observed associations in knowledge, attitudes, and practices. Moreover, the cross-sectional nature did not capture temporal variables such as when participants last updated their TEG knowledge or received training, which could influence KAP scores and partially explain the observed variance. Future longitudinal studies should incorporate such temporal measures to better understand changes over time. Fourth, the sample size limited our ability to conduct robust multi-group SEM comparisons across all professional subgroups, and departmental-level data (e.g., ICU vs. general ward) were not collected. These constraints prevented assessment of potential moderation effects of clinical setting on the KAP relationships. Fifth, we did not formally assess measurement invariance of the questionnaire across demographic subgroups before making between-group comparisons. Without establishing that the instrument measures the same constructs equivalently for all participants, group differences should be interpreted with caution. Sixth, we did not assess broader organizational culture variables, such as institutional support for innovation, error reporting culture, or resource availability, which may influence whether positive attitudes toward TEG translate into consistent practice. The absence of these measures may partially explain the limited direct relationship observed between knowledge and practice. Seventh, we did not incorporate constructs from Technology Acceptance Models, such as perceived usefulness and perceived ease of use, which could offer additional explanatory power for the observed attitude–practice gap. Including these constructs in future research could complement the KAP framework and provide a more comprehensive understanding of TEG adoption. Eighth, no formal algorithm for TEG application has been established in the study hospital, which may contribute to variability in clinical practice and limit the standardization of TEG use. Ninth, this study did not include a comparison between TEG and conventional coagulation tests (e.g., PT, APTT), which restricts the contextual understanding of how TEG is perceived and applied relative to standard assessments. Additionally, we did not collect data on participants’ clinical specialties, which may influence their perceptions and utilization of TEG. Future large-scale, multi-center, and longitudinal studies should aim to develop standardized TEG implementation protocols, assess their clinical impact, explore potential moderators such as clinical setting and organizational culture, and conduct comparative analyses with conventional coagulation tests to enhance the evidence base for its integration into routine practice.

In conclusion, the majority of healthcare professionals demonstrated inadequate knowledge, positive attitudes, and optimal practices regarding TEG, with significant correlations between these dimensions. To enhance TEG utilization, targeted training programs should be implemented, particularly for professionals with fewer years of experience and lower job ranks, to improve their proficiency and integration of TEG into clinical practice.

## Data Availability

The original contributions presented in the study are included in the article/Supplementary material, further inquiries can be directed to the corresponding authors.

## Footnotes

^1^ https://www.wjx.cn/

## References

[1] Othman M Kaur H. Thromboelastography (TEG). Methods Mol Biol. (2017) 1646:533–43. doi: 10.1007/978-1-4939-7196-1_39, PMID: 28804853

[2] Mallett SV. Clinical utility of viscoelastic tests of coagulation (TEG/ROTEM) in patients with liver disease and during liver transplantation. Semin Thromb Hemost. (2015) 41:527–37. doi: 10.1055/s-0035-1550434, PMID: 26049072

[3] Ramanujam V DiMaria S Varma V. Thromboelastography in the perioperative period: a literature review. Cureus. (2023) 15:e39407. doi: 10.7759/cureus.39407, PMID: 37362492 PMC10287184

[4] Liu J Liu Z Zhao T Su T Jin Q. Thromboelastography and traditional coagulation testing in non-ICU-admitted patients with acute kidney injury: an observational cohort study. Am J Nephrol. (2023) 54:208–18. doi: 10.1159/000530777, PMID: 37364534

[5] Yang L Ruan L Zhao Y Lu Y Shan Y Zhang Y. Association between thromboelastography and coronary heart disease. Med Sci Monit. (2022) 28:e935340. doi: 10.12659/MSM.935340, PMID: 35490293 PMC9069971

[6] Zhao H Cheng H Huang M Mei F. Application of thromboelastography in diagnosing normal pregnancies and pregnancies with complications. J Clin Lab Anal. (2022) 36:e24446. doi: 10.1002/jcla.24446, PMID: 35466451 PMC9169206

[7] Neal MD Moore HB Moore EE Freeman K Cohen MJ Sperry JL. Clinical assessment of trauma-induced coagulopathy and its contribution to postinjury mortality: a TACTIC proposal. J Trauma Acute Care Surg. (2015) 79:490–2. doi: 10.1097/TA.0000000000000793, PMID: 26307885 PMC5292045

[8] Gao L Su S Du N Han Y Wei J Cao M. Medical and non-medical students' knowledge, attitude and willingness towards the COVID-19 vaccine in China: a cross-sectional online survey. Hum Vaccin Immunother. (2022) 18:2073757. doi: 10.1080/21645515.2022.2073757, PMID: 35612817 PMC9359383

[9] Twinamasiko N Olum R Gwokyalya AM Nakityo I Wasswa E Sserunjogi E. Assessing knowledge, attitudes and practices towards COVID-19 public health preventive measures among patients at Mulago National Referral Hospital. Risk Manag Healthc Policy. (2021) 14:221–30. doi: 10.2147/RMHP.S287379, PMID: 33505175 PMC7829119

[10] Wang J Chen L Yu M He J. Impact of knowledge, attitude, and practice (KAP)-based rehabilitation education on the KAP of patients with intervertebral disc herniation. Ann Palliat Med. (2020) 9:388–93. doi: 10.21037/apm.2020.03.01, PMID: 32233633

[11] Cabana MD Rand CS Powe NR Wu AW Wilson MH Abboud PA. Why don't physicians follow clinical practice guidelines? A framework for improvement. JAMA. (1999) 282:1458–65. doi: 10.1001/jama.282.15.1458, PMID: 10535437

[12] Milanes Villa Dr Y Buscemi C. Improving clinicians’ knowledge of Thromboelastography: A quality improvement project. Miami, Florida, USA: Florida International University. (2022).

[13] Hartmann J Hermelin D Levy JH. Viscoelastic testing: an illustrated review of technology and clinical applications. Res Pract Thromb Haemost. (2023) 7:100031. doi: 10.1016/j.rpth.2022.100031, PMID: 36760779 PMC9903681

[14] Whitton TP Healy WJ. Review of thromboelastography (TEG): medical and surgical applications. Ther Adv Pulm Crit Care Med. (2023) 18:29768675231208426. doi: 10.1177/29768675231208426, PMID: 38107072 PMC10725099

[15] Bugaev N Como JJ Golani G Freeman JJ Sawhney JS Vatsaas CJ. Thromboelastography and rotational thromboelastometry in bleeding patients with coagulopathy: practice management guideline from the eastern Association for the Surgery of trauma. J Trauma Acute Care Surg. (2020) 89:999–1017. doi: 10.1097/TA.0000000000002944, PMID: 32941349

[16] Willis J Carroll C Planz V Galgano SJ. Thromboelastography: a review for radiologists and implications on periprocedural bleeding risk. Abdominal Radiology. (2022) 47:2697–703. doi: 10.1007/s00261-022-03539-9, PMID: 35567618 PMC9107068

[17] Schmidt AE Israel AK Refaai MA. The utility of thromboelastography to guide blood product transfusion: an ACLPS critical review. Am J Clin Pathol. (2019) 152:407–22. doi: 10.1093/ajcp/aqz074, PMID: 31263903

[18] Hebo HJ Gemeda DH Abdusemed KA. Hepatitis B and C viral infection: prevalence, knowledge, attitude, practice, and occupational exposure among healthcare workers of Jimma University Medical Center, Southwest Ethiopia. Sci World J. (2019) 2019:9482607. doi: 10.1155/2019/9482607, PMID: 30853866 PMC6377947

[19] Salman M Mustafa ZU Rao AZ Khan QU Asif N Hussain K. Serious inadequacies in high alert medication-related knowledge among Pakistani nurses: findings of a large, multicenter, cross-sectional survey. Front Pharmacol. (2020) 11:1026. doi: 10.3389/fphar.2020.01026, PMID: 32765259 PMC7381221

[20] Sun J Chen Y Wang M Dong N Qi D. Knowledge, attitude and practice of pediatric healthcare staff towards the therapy for patients with congenital heart disease. BMC Med Educ. (2024) 24:1312. doi: 10.1186/s12909-024-06305-1, PMID: 39543572 PMC11566178

[21] Zhao J Cao A Xie L Shao L. Knowledge, attitude, and practice toward oral health management among orthodontic patients: a cross-sectional study. BMC Oral Health. (2024) 24:1500. doi: 10.1186/s12903-024-05292-5, PMID: 39695598 PMC11658226

[22] Hosoi H Akagi Y Mushino T Takeyama M Minoura N Hiroi T. Use of thromboelastography before the administration of hemostatic agents to safely taper recombinant activated factor VII in acquired hemophilia a: a report of three cases. Thromb J. (2022) 20:28. doi: 10.1186/s12959-022-00387-x, PMID: 35578257 PMC9109301

[23] Aase I Akerjordet K Crookes P Frøiland CT Laugaland KA. Exploring the formal assessment discussions in clinical nursing education: an observational study. BMC Nurs. (2022) 21:155. doi: 10.1186/s12912-022-00934-x, PMID: 35710411 PMC9202123

[24] Montilla-Herrador J Lozano-Meca JA Baño-Alcaraz A Lillo-Navarro C Martín-San Agustín R Gacto-Sánchez M. Knowledge and attitudes towards patient safety among students in physical therapy in Spain: a longitudinal study. Int J Environ Res Public Health. (2022) 19:19. doi: 10.3390/ijerph191811618, PMID: 36141888 PMC9517046

[25] Tsantes AG Papadopoulos DV Trikoupis IG Tsante KA Mavrogenis AF Koulouvaris P. Rotational Thromboelastometry findings are associated with symptomatic venous thromboembolic complications after hip fracture surgery. Clin Orthop Relat Res. (2021) 479:2457–67. doi: 10.1097/CORR.0000000000001832, PMID: 34076610 PMC8509944

[26] Vaona A Banzi R Kwag KH Rigon G Cereda D Pecoraro V. E-learning for health professionals. Cochrane Database Syst Rev. (2018) 2018:CD011736. doi: 10.1002/14651858.CD011736.pub2, PMID: 29355907 PMC6491176

[27] Hossain MS Siam MHB Hasan MN Jahan R Siddiqee MH. Knowledge, attitude, and practice towards blood donation among residential students and teachers of religious institutions in Bangladesh–a cross-sectional study. Heliyon. (2022) 8:e10792. doi: 10.1016/j.heliyon.2022.e10792, PMID: 36203898 PMC9529581

[28] Franchini M Mengoli C Cruciani M Marietta M Marano G Vaglio S. The use of viscoelastic haemostatic assays in non-cardiac surgical settings: a systematic review and meta-analysis. Blood Transfus. (2018) 16:235. doi: 10.2450/2018.0003-18, PMID: 29517967 PMC5919835

[29] Hayes K Fernando MC Young L Jordan V. Prothrombin complex concentrate in cardiac surgery for the treatment of non-surgical bleeding. Cochrane Database Syst Rev. (2020) 2020:CD013551. doi: 10.1002/14651858.CD013551 PMC9677522 36408876

[30] Adler M Ivic S Bodmer NS Ten Cate H Bachmann LM Wuillemin WA. Thromboelastometry and thrombelastography analysis under normal physiological conditions-systematic review. Transfus Med Hemother. (2017) 44:78–83. doi: 10.1159/000464297, PMID: 28503123 PMC5425766

[31] Cannon JW Dias JD Kumar MA Walsh M Thomas SG Cotton BA. Use of thromboelastography in the evaluation and management of patients with traumatic brain injury: a systematic review and meta-analysis. Crit Care Explor. (2021) 3:e0526. doi: 10.1097/CCE.0000000000000526, PMID: 34549189 PMC8443808

[32] Marsee MK Shariff FS Wiarda G Watson PJ Sualeh AH Brenner TJ. Use of thromboelastography and rotational thromboelastometry in otolaryngology: a narrative review. J Clin Med. (2022) 11:1119. doi: 10.3390/jcm11041119, PMID: 35207392 PMC8876674

[33] Burton AG Jandrey KE. Use of thromboelastography in clinical practice. Vet Clin North Am Small Anim Pract. (2020) 50:1397–409. doi: 10.1016/j.cvsm.2020.08.001, PMID: 32981595

[34] Chen F Zhang L Bai X Wang X Geng Z. Clinical application of Thromboelastography in acute ischemic stroke. Clin Appl Thromb Hemost. (2022) 28:10760296221131801. doi: 10.1177/10760296221131801, PMID: 36285384 PMC9608017

[35] Subramanian M Kaplan LJ Cannon JW. Thromboelastography-guided resuscitation of the trauma patient. JAMA Surg. (2019) 154:1152–3. doi: 10.1001/jamasurg.2019.3136, PMID: 31596452

[36] Cheng D Li X Zhao S Hao Y. Establishment of thromboelastography reference intervals by indirect method and relevant factor analyses. J Clin Lab Anal. (2020) 34:e23224. doi: 10.1002/jcla.23224, PMID: 32004399 PMC7307360

[37] Sakai T. Comparison between thromboelastography and thromboelastometry. Minerva Anestesiol. (2019) 85:1346–56. doi: 10.23736/S0375-9393.19.13687-5, PMID: 31630507

[38] Vollmer NJ Leshko NA Wilson CS Gilbert BW. A review of thromboelastography for nurses. Crit Care Nurse. (2023) 43:29–37. doi: 10.4037/ccn2023371, PMID: 37257875

