# Supplementary material for: Healthcare professionals’ knowledge, attitudes and practices in thromboelastography application
Source: Front Med (Lausanne). 2025 Oct 7;12:1645570. doi: 10.3389/fmed.2025.1645570 (PMC12537656; doi:10.3389/fmed.2025.1645570)
Supplement: Supplementary file 1 [file Table_1.docx]

**Table S1. Mediation analyses**

| Model paths |  | Total effects | | Direct Effect | | Indirect effect | |
| --- | --- | --- | --- | --- | --- | --- | --- |
|  |  | β (95% CI) | P | β (95% CI) | P | β (95% CI) | P |
| Asum |  |  |  |  |  |  |  |
|  | Ksum | 0.76 (0.37, 1.14) | <0.001 | 0.76 (0.37, 1.14) | <0.001 |  |  |
|  | Job rank | 0.05 (-0.30, 0.42) | 0.749 |  |  | 0.05 (-0.30, 0.42) | 0.749 |
|  | Job years | 0.64 (0.10, 1.18) | 0.018 |  |  | 0.64 (0.10, 1.18) | 0.018 |
|  | **Has your hospital provided training related to thromboelastography** | -0.65 (-2.72, 1.40) | 0.531 | -0.65 (-2.72, 1.40) | 0.531 |  |  |
|  | **Frequently use thromboelastography in clinical practice** | -0.60 (-1.21, 0.00) | 0.051 |  |  | -0.60 (-1.21, 0.00) | 0.051 |
|  | Age | -0.15 (-0.75, 0.44) | 0.617 |  |  | -0.15 (-0.75, 0.44) | 0.617 |
| Psum |  |  |  |  |  |  |  |
|  | Asum | 1.10 (1.02, 1.19) | <0.001 | 1.10 (1.02, 1.19) | <0.001 |  |  |
|  | Ksum |  | <0.001 | 0.12 (-0.12, 0.37) | 0.335 | 0.84 (0.41, 1.27) | <0.001 |
|  | Job rank | -1.03 (-1.97, -0.09) | 0.03 | -1.11 (-1.93, -0.29) | 0.008 | 0.07 (-0.38, 0.53) | 0.749 |
|  | Job years | -0.70 (-1.76, 0.36) | 0.199 | -1.52 (-2.39, -0.64) | 0.001 | 0.81 (0.13, 1.50) | 0.018 |
|  | Education level | -0.34 (-1.54, 0.84) | 0.57 | -0.34 (-1.54, 0.84) | 0.57 |  |  |
|  | **Has your hospital provided training related to thromboelastography** | -0.73 (-3.01, 1.55) | 0.531 |  |  | -0.73 (-3.01, 1.55) | 0.531 |
|  | **Frequently use thromboelastography in clinical practice** | -0.77 (-1.54, 0.00) | 0.051 |  |  | -0.77 (-1.54, 0.00) | 0.051 |
|  | Age | -0.19 (-0.95, 0.56) | 0.617 |  |  | -0.19 (-0.95, 0.56) | 0.617 |
| Ksum |  |  |  |  |  |  |  |
|  | Job rank | 0.07 (-0.39, 0.55) | 0.748 | 0.07 (-0.39, 0.55) | 0.748 |  |  |
|  | Job years | 0.84 (0.28, 1.40) | 0.003 | 0.84 (0.28, 1.40) | 0.003 |  |  |
|  | **Frequently use thromboelastography in clinical practice** | -0.79 (-1.48, -0.10) | 0.024 | -0.79 (-1.48, -0.10) | 0.024 |  |  |
|  | Age | -0.20 (-0.97, 0.57) | 0.614 | -0.20 (-0.97, 0.57) | 0.614 |  |  |

**Table S2. Model fit indices**

| Indicators | Reference | Results |
| --- | --- | --- |
| RMSEA | <0.08 Good | 0.003 |
| SRMR | <0.08 Good | 0.033 |
| TLI | >0.8 Good | 1.000 |
| CFI | >0.8 Good | 1.000 |
