# Supplementary figures and images for: Healthcare professionals’ knowledge, attitudes and practices in thromboelastography application
Source: Front Med (Lausanne). 2025 Oct 7;12:1645570. doi: 10.3389/fmed.2025.1645570 (PMC12537656; doi:10.3389/fmed.2025.1645570)

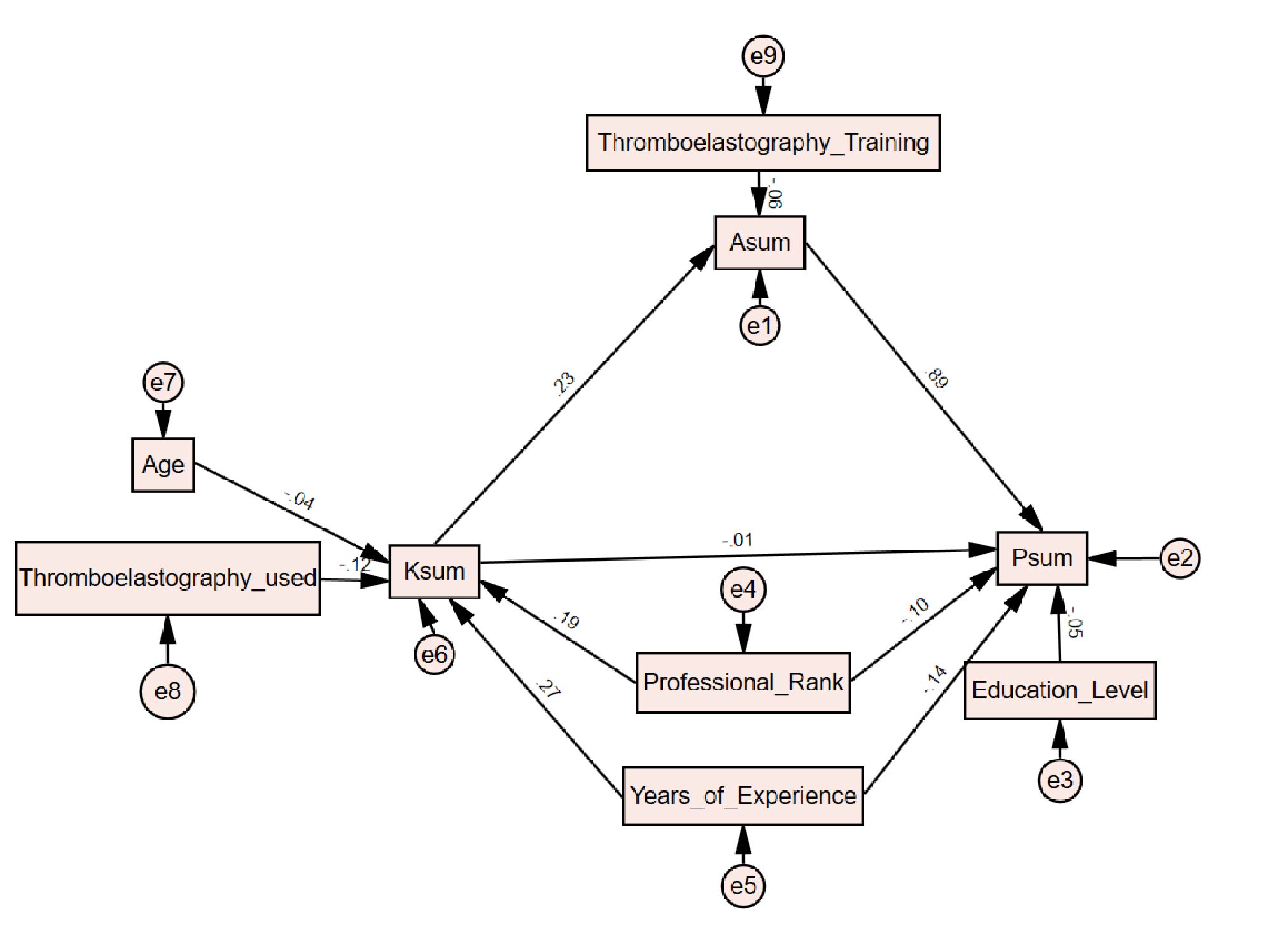

Supplement: Supplementary file 2 [file Image_1.jpeg]

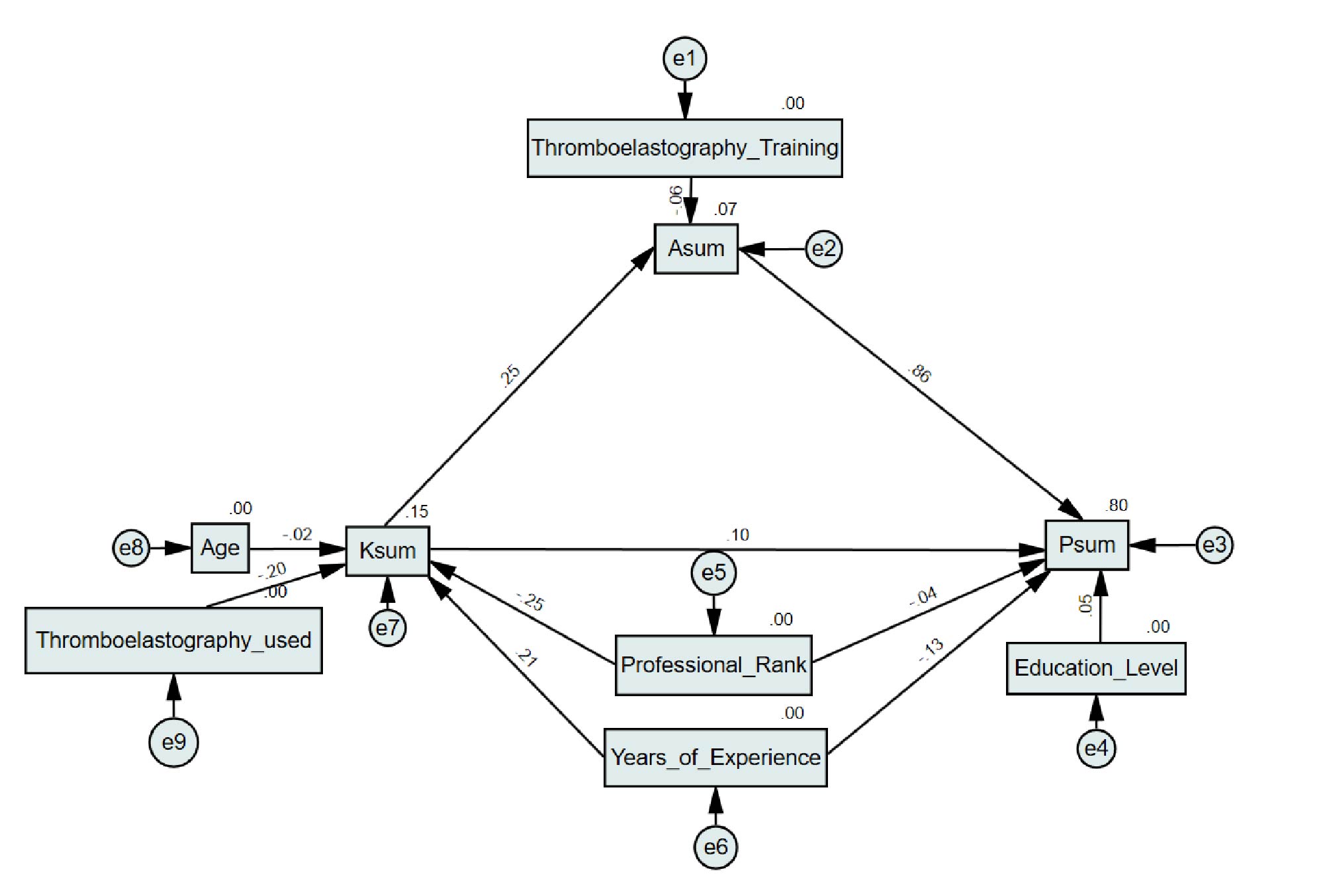

Supplement: Supplementary file 3 [file Image_2.jpeg]
